# Advances in sodium-glucose transporter protein 2 inhibitors and tumors

**DOI:** 10.3389/fonc.2025.1522059

**Published:** 2025-02-11

**Authors:** Jiaqi Wang, Wenyong Yang

**Affiliations:** ^1^ Department of Oncology, Guangyuan Central Hospital, Guangyuan, Sichuan, China; ^2^ Department of Respiratory and Critical Care Medicine, Guangyuan Central Hospital, Guangyuan, Sichuan, China

**Keywords:** SGLT2i, cancer, diabetic, chemotherapy side effects, carcinogenicity

## Abstract

Tumor is a major challenge to global health and has received extensive attention worldwide due to its high degree of malignancy and poor prognosis. Although the clinical application of targeted therapy and immunotherapy has improved the status quo of tumor treatment, the development of new therapeutic tools for tumors is still necessary. Sodium-glucose transporter protein 2 (SGLT2) inhibitors are a new type of glycemic control drugs, which are widely used in clinical practice because of their effects on weight reduction and protection of cardiac and renal functions. SGLT2 has been found to be overexpressed in many tumors and involved in tumorigenesis, progression and metastasis, suggesting that SGLT2i has a wide range of applications in tumor therapy. The aim of this article is to provide a comprehensive understanding of the research progress of SGLT2i in different tumors by integrating the latest studies and to encourage further exploration of SGLT2i therapies in clinical trials. This could pave the way for more effective management strategies and improved outcomes for tumor patients.

## 1 Introduction

Tumor is a major global public health problem, and the morbidity and mortality of this fatal disease are rapidly increasing. According to statistics, in 2020 alone, there will be approximately 19.3 million new cases of tumors and 10 million deaths due to tumors worldwide (1), bringing a serious economic burden to society and families. Although the mature application of targeted drugs and the emergence of immunotherapy provide more opportunities to reduce tumor deaths, drug resistance and disease recurrence remain long-term challenges. Therefore, the development of new drugs that can effectively control tumors is crucial. About a century ago, Warburg found that tumor cells exhibit unique metabolic characteristics, consuming more glucose than normal cells (2); reprogramming of glucose metabolism leads to several hallmark features of tumors, such as accelerated cell proliferation, angiogenesis, metastasis, and evasion of apoptosis (3). Epidemiological studies have shown that diabetic patients are at a significantly higher risk of developing tumors, and the presence of type 2 diabetes mellitus (T2DM) may increase the risk of cancer by 10%; hyperglycemia, hyperinsulinemia, genetic factors, inflammation, and oxidative stress are the links between diabetes and tumors (4). Given that cancer cell growth and proliferation are heavily dependent on glucose utilization, controlling tumor glucose metabolism is a very promising anti-tumor approach.

Sodium-glucose cotransporter (SGLT) belongs to the SLC5 family of active glucose transporter proteins, which can actively transport glucose against the concentration gradient by coupling with sodium; six different isoforms have been reported in humans, and SGLT1 and SGLT2 have been studied more frequently. SGLT1 is mainly responsible for glucose absorption in the small intestine, while SGLT2 is mainly responsible for glucose reabsorption in the kidneys, and more than 80% of the filtered glucose is reabsorbed through SGLT2 in the S1 and S2 segments of the proximal tubule, in addition to SGLT2, which is also expressed in the mammary glands, testis, liver, lungs, intestines, skeletal muscles, spleen, and cerebellum (5). SGLT2 inhibitors (SGLT2i) developed on the basis of phlorizin, which can inhibit SGLT1 and SGLT2, are new type of glycemic drugs, including canagliflozin, dapagliflozin, empagliflozin,tofogliflozin and so on, which can inhibit glucose reabsorption and increase urinary glucose excretion by acting on SGLT2 in the renal proximal tubule, and control blood glucose independently of the action of insulin. Further studies have shown that SGLT2 is expressed in a variety of tumor cells, and dapagliflozin significantly improves the survival rate of solid tumor models in mice (6). A meta-analysis also showed that SGLT2i was significantly associated with a reduced overall tumor risk compared with placebo (RR 0.35, CI 0.33-0.37, P < 0. 00) (7); suggesting that SGLT2i has a wide range of applications in tumor therapy. In this paper, we review the research progress of SGLT2i and tumor.

## 2 SGLT2i and hepatocellular carcinoma

Hepatocellular carcinoma (HCC) is the sixth most common cancer and the third leading cause of tumor-related deaths worldwide (8). Metabolic syndrome and obesity have been found to be key risk factors for hepatocellular carcinoma because obesity and T2DM significantly increase the incidence of nonalcoholic steatohepatitis (NASH) and nonalcoholic fatty liver disease (NAFLD),which are closely associated with increased risk of HCC, and thus intervention for NAFLD is important for the development of HCC. In diabetic and NASH/HCC mouse models, both canagliflozin and tofogliflozin exhibited anti-steatosis and anti-inflammatory effects, attenuated the development of NASH and prevented the progression of NASH to HCC (9–11), so SGLT2i may have a chemopreventive effect on hepatocellular carcinoma associated with T2DM, obesity and NAFLD. In addition, canagliflozin inhibited glucose uptake, β-cyclin activation, lactate release and angiogenic activity, restored PDH activity, increased intracellular ROS, consumed intracellular ATP and regulated endoplasmic reticulum stress-mediated cytoprotective autophagy, which was converted to cytotoxic autophagy and apoptosis, and also inhibited HIF-1α protein, which reduced metastasis, angiogenesis, and thus attenuated hepatocellular carcinoma growth (12–15). Multi-omics analysis of metabolomics and absolute quantitative proteomics showed that canagliflozin could alter the phosphorylation of AMPK and ACC, and inhibit the value-added of hepatocellular carcinoma cells by regulating the electron transport system, β-oxidation and nucleic acid synthesis (16). Empagliflozin could inhibit the ability of MAPK, p38, and ERK1/2, and enhance the antimetabolic effect of metformin by controlling the angiogenesis and metastasis better. enhance the anti-tumor function of metformin (17). A study based on the large SEER-Medicare database found that patients with prior T2DM and hepatocellular carcinoma treated with SGLT2i had a significantly lower risk of death, especially those treated for more than 12 months (18). In addition, canagliflozin induces iron death through its dual effect on glycolysis and glutamine metabolism, re-sensitizing hepatocellular carcinoma to cisplatin and increasing sensitivity to chemotherapy (19). However, a recent study showed that high concentrations of trilobatin (an analogue of phlorizin) accelerated the proliferation of human hepatoblastoma HepG2 cells, but not that of the human normal hepatocyte cell line LO2 and other cells (20). In conclusion, the current study is more limited to animal models and retrospective analyses, more clinical trials are needed, and it should be alerted whether high concentrations of SGLT2i promote tumor progression.

## 3 SGLT2i and lung cancer

The putative role of SGLT2 in lung cancer metastasis was first reported by Ishikawa et al. They found that there was no significant difference in the expression of SGLT2 in primary lung cancer lesions and normal tissues, but the expression of SGLT2 in liver and lymph node metastatic lesions was significantly higher than that in primary lesions, and these results led to the preliminary speculative conclusion that SGLT2 plays a key role in glucose uptake in lung cancer metastasis (21). With the wide application of SGLT2i in T2DM, the prospect of its application in lung cancer has received increasing attention. It has been found that down-regulation of SGLT2 can limit the growth of non-small cell lung cancer (NSCLC) (22), while canagliflozin can induce apoptosis in NSCLC cells harboring the EGFR T790M mutation (23). A large SEER-Medicare linked data study showed that the use of SGLT2i was associated with improved overall survival in diabetic patients with NSCLC (24). However, in another study, although SGLT2 expression was found in human lung cancer tissues and cell lines, and *in vitro* experiments showed that canagliflozin attenuated lung cancer cell proliferation and DNA synthesis by inhibiting cell cycle progression, no reduction in tumor growth was found in *in vivo* experiments (25). Scafoglio CR et al. found that SGLT2 was mainly expressed in precancerous lesions and early, well-differentiated lung adenocarcinoma;in mouse experiments, it was shown that the tumor proliferation rate of the canagliflozin treatment group was significantly reduced, but only for precancerous lesions, not for solid lung adenocarcinoma, indicating that SGLT2i may target precancerous lesions and its efficacy is higher than that of advanced tumors, suggesting that SGLT2 is a diagnostic and therapeutic target for early-stage lung adenocarcinoma (26). Therefore, SGLT2i may contribute to the prevention and early treatment of lung cancer, while its application in advanced tumors requires further clinical studies.

## 4 SGLT2i and breast cancer

Breast cancer is a major health problem for women because of its high mortality and morbidity. It has been reported that in 2020, there will be more than 2 million newly diagnosed breast cancer patients globally, of which 685,000 will die from the disease, another one-quarter of women will develop breast cancer, and one-eighth of women will die from breast cancer. The five-year survival rate for metastatic breast cancer, even with adjuvant chemotherapy, is less than 30% (1, 27). Therefore, it is important to explore new therapeutic approaches for breast cancer. Although a recent retrospective study showed no significant correlation between SGLT2 levels and clinical outcomes (28), the findings of SGLT2i in breast cancer are exciting enough. It was found that SGLT2i could attenuate the proliferation of breast cancer cells by decreasing the effects of glucose and insulin, AMPK-mediated cell cycle arrest and apoptosis, membrane hyperpolarization and mitochondrial membrane instability, as well as interrupting mTOR-mediated inflammatory signaling (29–32); empagliflozin inactivates SP1 and PKM2 by enhancing the expression of miR-128-3p, thus attenuating breast cancer proliferation (33); canagliflozin also inhibits breast cancer cell proliferation by reducing oxygen consumption and glutamine metabolism through the citric acid cycle, and its antiproliferative effect is not affected by glucose utilization or SGLT2 expression level (34). Therefore, SGLT2i may not control tumor growth through the inhibition of glucose uptake by SGLT2, but through various other pathways to control the growth of breast cancer, but its clinical application needs to be further investigated.

## 5 SGLT2i and pancreatic cancer

Pancreatic cancer is a highly aggressive malignant tumor with a 5-year overall survival rate of less than 6% and a median survival period of 3-6 months (35). Most pancreatic cancers are already in advanced stages when diagnosed, losing the chance of surgery, and radiotherapy and chemotherapy have not yet brought satisfactory results to pancreatic cancer patients; therefore, it is necessary to find new treatment methods. It has been found that SGLT2 is functionally expressed in pancreatic cancer, and SGLT2 can activate the Hippo signaling pathway through the hnRNPK-YAP1 axis to promote the progression of pancreatic cancer (36, 37). Furthermore, data from the Cancer Genome Atlas showed that high SGLT2 expression coexisted with impaired cellular replicative surveillance, enhanced cellular metabolism and drug metabolism, and that high levels of SGLT2 coexisted with up-regulation of gene signatures in pancreatic progenitor cell subsets in pancreatic cancer (38). *In vitro* studies have demonstrated that canagliflozin inhibits pancreatic cancer growth by down-regulating GLUT-1 and LDHA and inhibiting glycolysis through the PI3K/AKT/mTOR/HIF-1α signaling pathway at both the mRNA and protein levels, and the combination of this treatment with gemcitabine has shown improved therapeutic efficacy in pancreatic cancer (39). In a recent phase 1b clinical trial in patients with newly diagnosed advanced pancreatic cancer, dapagliflozin was well tolerated and safe, and had a beneficial effect on advanced pancreatic cancer as an adjuvant to standard GP chemotherapy (40). However, retrospective analysis showed that SGLT2 was not significantly expressed in pancreatic cancer, but SGLT1 was significantly overexpressed; the median overall and progression-free survival of patients with high expression of SGLT1 was significantly longer than that of patients with low expression of SGLT1, suggesting that high expression of SGLT1 is an independent predictor of better prognosis (41). Therefore, SGLT2i also exists for pancreatic cancer similar to breast cancer, inhibiting tumor growth not through SGLT2 but through other pathways, but larger clinical studies are needed.

## 6 SGLT2 inhibitors and urologic tumors

Bladder cancer is the most common tumor of the urinary tract, and a case analysis of the European Pharmacovigilance Database showed that the number of bladder cancer cases was abnormally high in patients using SGLT2i (42); however, dapagliflozin has not been found to act as a promoter or progression of bladder cancer in a rat model of bladder cancer (43); a retrospective study in Taiwan demonstrated that the combination of SGLT2i and pioglitazone was not associated with new diagnosis of bladder cancer and had a low all-cause mortality rate for patients with no previous or active bladder cancer (44); an international multicenter cohort study showed that SGLT2i and pioglitazone were not associated with new diagnosis and had a lower all-cause mortality rate in T2DM patients without prior or active bladder cancer (44); an international multicenter cohort study showed that the use of SGLT2i was not associated with an increased risk of bladder cancer compared with glucagon-like peptide 1 receptor agonist or dipeptide kineptinase 4 inhibitor(DPP4i) (45). Therefore, the association between SGLT2i and bladder cancer should be taken seriously, but it is not a cause for concern. Prostate cancer is a common disease in elderly men, with the highest incidence rate in Europe and the United States, and its incidence rate has exceeded that of lung cancer, becoming the number one tumor endangering men’s health. Studies have found that SGLT2 is functionally expressed in prostate cancer and that canagliflozin inhibits mitochondrial complex I-supported respiration and cell proliferation in prostate cancer cells to reduce cancer cell proliferation and enhance their sensitivity to radiotherapy (36, 46, 47), providing a basis for the use of SGLT2i in prostate cancer. However, a recent meta-analysis including randomized controlled trials with a follow-up time of more than 2 years showed that SGLT2i did not have significant oncogenic and preventive effects on prostate cancer (OR = 1.17, 95% CI: 0.87-1.57) (48), therefore the relationship between SGLT2i and prostate cancer needs to be further investigated, and more clinical and *in vivo* trials are needed. Renal cell carcinoma (RCC) accounts for about 2% of all human malignant tumors; it is more common in males than in females, and clear cell type is its main component, accounting for about 70-80%. In an *in vitro* study, the expression level of SGLT2 in renal cancer cell lines was found to be significantly higher than that in normal cells, and dapagliflozin could inhibit tumor growth by reducing the viability of renal cancer cell lines and regulating the cell cycle and apoptosis (49); a study in Japan showed that the expression of SGLT2 in clear-cell carcinoma was significantly correlated with a shorter overall survival, regardless of metastatic status (p < 0.01) (50). Therefore, SGLT2i has a promising application in renal cell carcinoma, but more clinical studies are needed.

## 7 SGLT2 inhibitors and gastrointestinal tumors

Globally, colorectal cancer is the third most common cancer and the fourth leading cause of cancer-related deaths in men, and the second and third in women, respectively (51). An *in vitro* study found that dapagliflozin significantly reduced colorectal cancer cells (52), while in mouse experiments, tofogliflozin inhibited colorectal cancer development by reducing chronic inflammation induced by obesity and diabetes mellitus (53); a retrospective cohort study demonstrated that the use of SGLT2i reduced the risk of colorectal cancer in comparison with the use of DPP4i in T2DM patients (HR:0.526; 95% CI: 0.382-0.724; P<0.001), especially in young men with preserved renal function (54). A meta-analysis suggested that canagliflozin may have a preventive effect on gastrointestinal cancers (OR 0.15 [95% CI 0.04,0.60]) (55), and the mechanism may be that canagliflozin can inhibit HDAC6, which can inhibit gastric cancer metastasis (56). There is a case report that dapagliflozin combined with cetuximab significantly reduced tumor size and CEA level in sigmoid colon cancer liver metastasis cases (57). However, another study found that, compared with placebo, empagliflozin may increase the overall risk of malignant tumors, mainly of the digestive system (58). Therefore, different SGLT2i may exhibit different effects on gastrointestinal tumors; the exact relationship needs further investigation.

## 8 SGLT2 inhibitors and other tumors

SGLT2i was found to attenuate the growth of thyroid cancer cells *in vitro* and *in vivo*, and its mechanism may include 1) inhibiting the level of glucose uptake and glycolysis, suppressing the activation of AKT/mTOR, and increasing the activation of AMPK, which inhibited the growth of thyroid cancer cells. This resulted in decreased proliferation of thyroid cancer; 2) induced ROS-mediated DNA damage and activation of ATM/CHK2, leading to the arrest of G1/S phase transition and increased apoptosis in thyroid cancer (59). In the study of cervical cancer, it was found that empagliflozin could activate AMPK phosphorylation, down-regulate the expression of FOXA1, and thus inhibit the expression of SHH, inhibit the malignant proliferation of cervical cancer cells and induce apoptosis (60). Wu et al. found that SGLT2 was overexpressed in osteosarcoma, and SGLT2i significantly inhibited osteosarcoma tumor growth and induced immune cell infiltration *in vivo* by up-regulating the expression of STING and activating the IRF3/IFN-β pathway (61). In addition, SGLT2i can inhibit the growth of adult T-cell leukemia cells by attenuating glucose uptake and decreasing intracellular ATP and NADPH levels (62), and canagliflozin can inhibit the growth and proliferation of glioblastomas by activating AMPK in animal experiments (63), so SGLT2i has the potential to be widely applied to various types of tumors. More clinical studies are expected.

## 9 SGLT2i and chemotherapeutic agents

With the progress of medical technology, more and more therapeutic methods have been applied to the treatment of tumors, and the status of targeted therapy and tumor immunotherapy has been greatly enhanced; however, chemotherapy still has a pivotal position as the basis of antitumor therapy, but its adverse effects limit its further application; it has been found that SGLT2i can significantly attenuate the adverse effects of a variety of chemotherapeutic drugs; empagliflozin can reduce trastuzumab cardiotoxicity by inhibiting DNA damage and iron death to reduce the cardiotoxicity of trastuzumab (64), improve sunitinib-induced cardiac dysfunction by regulating AMPK-mTOR signaling pathway-mediated autophagy in cardiomyocytes (65), and ameliorate adriamycin-induced acute cardiotoxicity (66). Dapagliflozin can significantly reduce cyclophosphamide-induced cardiotoxicity by modulating SGLT2 and HIF1α/HIF1α/eNOS signaling pathways (67), and also inhibit ER stress to reduce adriamycin-induced cardiomyocyte apoptosis (68). A meta-analysis showed that SGLT2i was effective in preventing anthracycline chemotherapy-induced left ventricular dysfunction in a mouse model (69), and retrospective analyses showed that SGLT2i was associated with a reduced incidence of cardiac events in cancer and diabetic patients treated with anthracyclines (70). However, canagliflozin had no significant effect on piroxicam-induced cardiotoxicity (71). Canagliflozin also prevented cisplatin-induced renal injury through anti-inflammatory, antioxidant, and AMPK-mediated autophagy in renal proximal tubular cells (72, 73). Empagliflozin also ameliorated bleomycin-induced pulmonary fibrosis (74).

## 10 SGLT2i and carcinogenicity

The carcinogenic potential of canagliflozin was identified in a 2-year rat study in which three tumors potentially associated with canagliflozin were observed: renal tubular tumors, pheochromocytomas, and testicular mesenchymal cell tumors; however, a follow-up study found that pheochromocytomas and renal tubular tumors were secondary to carbohydrate malabsorption and were not directly related to the exposure to canagliflozin; whereas, rats are thought to be genetically predisposed to testicular mesenchymal cell tumors, and the incidence of this tumor type is very low in humans, and T2DM subjects treated with clinical doses of 100 mg and 300 mg daily for 12 weeks with luteinizing hormone- or hormone-promoting hormone, were not considered to be carcinogenic. Considering that increased luteinizing hormone is an established mechanism common to many of the non-genotoxic drugs that cause testicular mesenchymal cell tumors in rats, and that no changes in luteinizing hormone or testosterone levels were observed in the T2DM subjects treated with canagliflozin at the clinical doses of 100 mg and 300 mg once a day for 12 weeks, and that the increase in luteinizing hormone is an established mechanism common to many of the non-genotoxic drugs that cause testicular mesenchymal cell tumors in rats, the observed changes in the levels of testicular mesenchymal tumors in canagliflozin-treated rats are not directly related to the exposure of canagliflozin. The testicular mesenchymal cell tumor formation observed in rats treated with canagliflozin is not considered clinically relevant (75–77). In toxicological studies of empagliflozin, testicular mesenchymal stromal cell tumors and mesenteric lymph node hemangiomas have been observed in male rats, and tubular adenomas and renal carcinomas may be induced in mice; however, the increased incidence of mesenchymal cell tumors is thought to be secondary to the significant decrease in body weight gain of treated males, while mesenteric lymph node hemangiomas in rats appear to be a species-specific and relatively common spontaneous tumor. spontaneous tumors, a finding considered to be of low safety relevance in humans; in addition to this, the effect of early empagliflozin-associated degenerative/regenerative changes observed only in high-dose male CD-1 mice, the observed renal tumors in male mice are therefore considered to be unrelated to humans (78–80). A study found that canagliflozin prolonged the lifespan of genetically heterozygous male but not female mice (81), which cannot be ruled out because canagliflozin increases the intestinal adenoma burden in female mice (82). Further studies on the effect of SGLT2i on gender variability are needed.

## 11 Conclusion

Although great progress has been made in the treatment of tumors, especially with the support of targeted drugs and tumor immunotherapy, it is still the second leading cause of death in the world. With the finding that SGLT2 is expressed in many different tumors, SGLT2i shows great potential for tumor treatment; a large number of *in vitro* studies have shown that SGLT2i can inhibit tumor growth and improve prognosis through multiple pathways independent of glucose-lowering effects, as well as reduce the side-effects of chemotherapeutic drugs and enhance the sensitivity, so there is a great potential for the application of SGLT2i. Considering that the important limitations of these drugs are their safety and clinical feasibility in tumor therapy, more comprehensive studies or clinical trials to explore the application of these exciting new antidiabetic drugs in oncology are necessary.

## References

[1] Sung H Ferlay J Siegel RL Laversanne M Soerjomataram I Jemal A. Global cancer statistics 2020: GLOBOCAN estimates of incidence and mortality worldwide for 36 cancers in 185 countries. CA Cancer J Clin. (2021) 71:209–49. doi: 10.3322/caac.21660 33538338

[2] Gyamfi J Kim J Choi J. Cancer as a metabolic disorder. Int J Mol Sci. (2022) 23:1155. doi: 10.3390/ijms23031155 35163079 PMC8835572

[3] Hanahan D Weinberg RA. Hallmarks of cancer: the next generation. Cell. (2011) 144:646–74. doi: 10.1016/j.cell.2011.02.013 21376230

[4] Zhu B Qu S. The relationship between diabetes mellitus and cancers and its underlying mechanisms. Front Endocrinol (Lausanne). (2022) 13. doi: 10.3389/fendo.2022.800995 PMC8873103 35222270

[5] Sano R Shinozaki Y Ohta T. Sodium-glucose cotransporters: Functional properties and pharmaceutical potential. J Diabetes Investig. (2020) 11:770–82. doi: 10.1111/jdi.13255 PMC7378437 32196987

[6] Kabel AM Arab HH Abd Elmaaboud MA. Effect of dapagliflozin and/or L-arginine on solid tumor model in mice: The interaction between nitric oxide, transforming growth factor-beta 1, autophagy, and apoptosis. Fundam Clin Pharmacol. (2021) 35:968–78. doi: 10.1111/fcp.12661 33609317

[7] Benedetti R Benincasa G Glass K Chianese U Vietri MT Congi R. Effects of novel SGLT2 inhibitors on cancer incidence in hyperglycemic patients: a meta-analysis of randomized clinical trials. Pharmacol Res. (2022) 175:106039. doi: 10.1016/j.phrs.2021.106039 34929299

[8] Forner A Reig M Bruix J. Hepatocellular carcinoma. Lancet. (2018) 391:1301–14. doi: 10.1016/S0140-6736(18)30010-2 29307467

[9] Shiba K Tsuchiya K Komiya C Miyachi Y Mori K Shimazu N. Canagliflozin, an SGLT2 inhibitor, attenuates the development of hepatocellular carcinoma in a mouse model of human NASH. Sci Rep. (2018) 8:2362. doi: 10.1038/s41598-018-19658-7 29402900 PMC5799179

[10] Jojima T Wakamatsu S Kase M Iijima T Maejima Y Shimomura K. The SGLT2 inhibitor canagliflozin prevents carcinogenesis in a mouse model of diabetes and non-alcoholic steatohepatitis-related hepatocarcinogenesis: association with SGLT2 expression in hepatocellular carcinoma. Int J Mol Sci. (2019) 20:5237. doi: 10.3390/ijms20205237 31652578 PMC6829338

[11] Obara K Shirakami Y Maruta A Ideta T Miyazaki T Kochi T. Preventive effects of the sodium glucose cotransporter 2 inhibitor tofogliflozin on diethylnitrosamine-induced liver tumorigenesis in obese and diabetic mice. Oncotarget. (2017) 8:58353–63. doi: 10.18632/oncotarget.16874 PMC5601657 28938561

[12] Hung MH Chen YL Chen LJ Chu PY Hsieh FS Tsai MH. Canagliflozin inhibits growth of hepatocellular carcinoma via blocking glucose-influx-induced β-catenin activation. Cell Death Dis. (2019) 10:420. doi: 10.1038/s41419-019-1646-6 31142735 PMC6541593

[13] Kaji K Nishimura N Seki K Sato S Saikawa S Nakanishi K. Sodium glucose cotransporter 2 inhibitor canagliflozin attenuates liver cancer cell growth and angiogenic activity by inhibiting glucose uptake. Int J Cancer. (2018) 142:1712–22. doi: 10.1002/ijc.31193 29205334

[14] Abdel-Rafei MK Thabet NM Rashed LA Moustafa EM. Canagliflozin, a SGLT-2 inhibitor, relieves ER stress, modulates autophagy and induces apoptosis in irradiated HepG2 cells: Signal transduction between PI3K/AKT/GSK-3β/mTOR and Wnt/β-catenin pathways; *in vitro* . J Cancer Res Ther. (2021) 17:1404–18. doi: 10.4103/jcrt.JCRT_963_19 34916371

[15] Luo J Sun P Zhang X Lin G Xin Q Niu Y. Canagliflozin modulates hypoxia-induced metastasis, angiogenesis and glycolysis by decreasing HIF-1α Protein synthesis via AKT/mTOR pathway. Int J Mol Sci. (2021) 22:13336. doi: 10.3390/ijms222413336 34948132 PMC8704642

[16] Nakano D Kawaguchi T Iwamoto H Hayakawa M Koga H Torimura T. Effects of canagliflozin on growth and metabolic reprograming in hepatocellular carcinoma cells: Multi-omics analysis of metabolomics and absolute quantification proteomics (iMPAQT). PloS One. (2020) 15:e0232283. doi: 10.1371/journal.pone.0232283 32343721 PMC7188283

[17] Abdelhamid AM Saber S Youssef ME Gaafar AGA Eissa H Abd-Eldayem MA. Empagliflozin adjunct with metformin for the inhibition of hepatocellular carcinoma progression: Emerging approach for new application. BioMed Pharmacother. (2022) 145:112455. doi: 10.1016/j.biopha.2021.112455 34844106

[18] Hendryx M Dong Y Ndeke JM Luo J. Sodium-glucose cotransporter 2 (SGLT2) inhibitor initiation and hepatocellular carcinoma prognosis. PloS One. (2022) 17:e0274519. doi: 10.1371/journal.pone.0274519 36094949 PMC9467321

[19] Zeng Y Jiang H Zhang X Xu J Wu X Xu Q. Canagliflozin reduces chemoresistance in hepatocellular carcinoma through PKM2-c-Myc complex-mediated glutamine starvation. Free Radic Biol Med. (2023) 208:571–86. doi: 10.1016/j.freeradbiomed.2023.09.006 37696420

[20] Wang L Liu M Yin F Wang Y Li X Wu Y. Trilobatin, a novel SGLT1/2 inhibitor, selectively induces the proliferation of human hepatoblastoma cells. Molecules. (2019) 24:3390. doi: 10.3390/molecules24183390 31540429 PMC6767144

[21] Ishikawa N Oguri T Isobe T Fujitaka K Kohno N. SGLT gene expression in primary lung cancers and their metastatic lesions. Jpn J Cancer Res. (2001) 92:874–9. doi: 10.1111/j.1349-7006.2001.tb01175.x PMC5926833 11509120

[22] Zhang X Zhang X Liu X Qi P Wang H Ma Z. MicroRNA-296, a suppressor non-coding RNA, downregulates SGLT2 expression in lung cancer. Int J Oncol. (2019) 54:199–208. doi: 10.3892/ijo.2018.4599 30365049

[23] Li H Tong CW Leung Y Wong MH To KK Leung KS. Identification of clinically approved drugs indacaterol and canagliflozin for repurposing to treat epidermal growth factor tyrosine kinase inhibitor-resistant lung cancer. Front Oncol. (2017) 7:288. doi: 10.3389/fonc.2017.00288 29238696 PMC5712561

[24] Luo J Hendryx M Dong Y. Sodium-glucose cotransporter 2 (SGLT2) inhibitors and non-small cell lung cancer survival. Br J Cancer. (2023) 128:1541–7. doi: 10.1038/s41416-023-02177-2 PMC10070339 36765176

[25] Yamamoto L Yamashita S Nomiyama T Kawanami T Hamaguchi Y Shigeoka T. Sodium-glucose cotransporter 2 inhibitor canagliflozin attenuates lung cancer cell proliferation *in vitro* . Diabetol Int. (2021) 12:389–98. doi: 10.1007/s13340-021-00494-6 PMC8413406 34567921

[26] Scafoglio CR Villegas B Abdelhady G Bailey ST Liu J Shirali AS. Sodium-glucose transporter 2 is a diagnostic and therapeutic target for early-stage lung adenocarcinoma. Sci Transl Med. (2018) 10:eaat5933. doi: 10.1126/scitranslmed.aat5933 30429355 PMC6428683

[27] Kashyap D Pal D Sharma R Garg VK Goel N Koundal D. Global increase in breast cancer incidence: risk factors and preventive measures. BioMed Res Int. (2022) 2022:9605439. doi: 10.1155/2022/9605439 35480139 PMC9038417

[28] Tsunokake S Iwabuchi E Miki Y Kanai A Onodera Y Sasano H. SGLT1 as an adverse prognostic factor in invasive ductal carcinoma of the breast. Breast Cancer Res Treat. (2023) 201:499–513. doi: 10.1007/s10549-023-07024-9 37439959

[29] Nasiri AR Rodrigues MR Li Z Leitner BP Perry RJ. SGLT2 inhibition slows tumor growth in mice by reversing hyperinsulinemia. Cancer Metab. (2019) 7:10. doi: 10.1186/s40170-019-0203-1 31867105 PMC6907191

[30] Zhou J Zhu J Yu SJ Ma HL Chen J Ding XF. Sodium-glucose co-transporter-2 (SGLT-2) inhibition reduces glucose uptake to induce breast cancer cell growth arrest through AMPK/mTOR pathway. BioMed Pharmacother. (2020) 132:110821. doi: 10.1016/j.biopha.2020.110821 33068934

[31] Komatsu S Nomiyama T Numata T Kawanami T Hamaguchi Y Iwaya C. SGLT2 inhibitor ipragliflozin attenuates breast cancer cell proliferation. Endocr J. (2020) 67:99–106. doi: 10.1507/endocrj.EJ19-0428 31776304

[32] Sabaa M Sharawy MH El-Sherbiny M Said E Salem HA Ibrahim TM. Canagliflozin interrupts mTOR-mediated inflammatory signaling and attenuates DMBA-induced mammary cell carcinoma in rats. BioMed Pharmacother. (2022) 155:113675. doi: 10.1016/j.biopha 36115110

[33] Nalla LV Khairnar A. Empagliflozin mediated miR-128-3p upregulation promotes differentiation of hypoxic cancer stem-like cells in breast cancer. Eur J Pharmacol. (2023) 943:175565. doi: 10.1016/j.ejphar.2023.175565 36739077

[34] Papadopoli D Uchenunu O Palia R Chekkal N Hulea L Topisirovic I. Perturbations of cancer cell metabolism by the antidiabetic drug canagliflozin. Neoplasia. (2021) 23:391–9. doi: 10.1016/j.neo.2021.02.003 PMC8027095 33784591

[35] Bray F Ferlay J Soerjomataram I Siegel RL Torre LA Jemal A. Global cancer statistics 2018: GLOBOCAN estimates of incidence and mortality worldwide for 36 cancers in 185 countries. CA Cancer J Clin. (2018) 68:394–424. doi: 10.3322/caac.21492 30207593

[36] Scafoglio C Hirayama BA Kepe V Liu J Ghezzi C Satyamurthy N. Functional expression of sodium-glucose transporters in cancer. Proc Natl Acad Sci U S A. (2015) 112:E4111–9. doi: 10.1073/pnas.1511698112 PMC4522748 26170283

[37] Ren D Sun Y Zhang D Li D Liu Z Jin X. SGLT2 promotes pancreatic cancer progression by activating the Hippo signaling pathway via the hnRNPK-YAP1 axis. Cancer Lett. (2021) 519:277–88. doi: 10.1016/j.canlet.2021.07.035 34314754

[38] Qiang W Lei Y Yuan L Yuan J Zhang J Shan Y. SGLT-2 as a potential target in pancreatic cancer: the preliminary clue from The Cancer Genome Atlas data. J Gastrointest Oncol. (2022) 13:2539–52. doi: 10.21037/jgo-22-900 PMC9660074 36388652

[39] Xu D Zhou Y Xie X He L Ding J Pang S. Inhibitory effects of canagliflozin on pancreatic cancer are mediated via the downregulation of glucose transporter 1 and lactate dehydrogenase A. Int J Oncol. (2020) 57:1223–33. doi: 10.3892/ijo.2020.5120 32901837

[40] Park LK Lim KH Volkman J Abdiannia M Johnston H Nigogosyan Z. Safety, tolerability, and effectiveness of the sodium-glucose cotransporter 2 inhibitor (SGLT2i) dapagliflozin in combination with standard chemotherapy for patients with advanced, inoperable pancreatic adenocarcinoma: a phase 1b observational study. Cancer Metab. (2023) 11:6. doi: 10.1186/s40170-023-00306-2 37202813 PMC10193807

[41] Du J Gu J Deng J Kong L Guo Y Jin C. The expression and survival significance of sodium glucose transporters in pancreatic cancer. BMC Cancer. (2022) 22:116. doi: 10.1186/s12885-021-09060-4 35090421 PMC8796473

[42] García M Arteche-Martinez U Lertxundi U Aguirre C. SGLT2 inhibitors and bladder cancer: analysis of cases reported in the European pharmacovigilance database. J Clin Pharmacol. (2021) 61:187–92. doi: 10.1002/jcph.1722 32827151

[43] Billger M Kirk J Chang J Bédard A Attalla B Haile S. A study in a rat initiation-promotion bladder tumour model demonstrated no promoter/progressor potential of dapagliflozin. Regul Toxicol Pharmacol. (2019) 103:166–73. doi: 10.1016/j.yrtph.2019.01.031 30685222

[44] Li YR Liu CH Sun WC Fan PY Liu FH Chen TH. The risk of bladder cancer in type 2 diabetes mellitus with combination therapy of SGLT-2 inhibitors and pioglitazone. J Pers Med. (2021) 11:828. doi: 10.3390/jpm11090828 34575605 PMC8472235

[45] Abrahami D Tesfaye H Yin H Vine S Hicks B Yu OHY. Sodium-glucose cotransporter 2 inhibitors and the short-term risk of bladder cancer: an international multisite cohort study. Diabetes Care. (2022) 45:2907–17. doi: 10.2337/dc22-1174 PMC9998845 36170656

[46] Villani LA Smith BK Marcinko K Ford RJ Broadfield LA Green AE. The diabetes medication Canagliflozin reduces cancer cell proliferation by inhibiting mitochondrial complex-I supported respiration. Mol Metab. (2016) 5:1048–56. doi: 10.1016/j.molmet.2016.08.014 PMC5034684 27689018

[47] Ali A Mekhaeil B Biziotis OD Tsakiridis EE Ahmadi E Wu J. The SGLT2 inhibitor canagliflozin suppresses growth and enhances prostate cancer response to radiotherapy. Commun Biol. (2023) 6:919. doi: 10.1038/s42003-023-05289-w 37684337 PMC10491589

[48] Cui H Wang Y Yang S He G Jiang Z Gang X. Antidiabetic medications and the risk of prostate cancer in patients with diabetes mellitus: A systematic review and meta-analysis. Pharmacol Res. (2022) 177:106094. doi: 10.1016/j.phrs.2022.106094 35074527

[49] Kuang H Liao L Chen H Kang Q Shu X Wang Y. Therapeutic effect of sodium glucose co-transporter 2 inhibitor dapagliflozin on renal cell carcinoma. Med Sci Monit. (2017) 23:3737–45. doi: 10.12659/msm.902530 PMC5549715 28763435

[50] Kobayashi M Uematsu T Tokura Y Takei K Sakamoto K Narimatsu T. Immunohistochemical expressionof sodium-dependent glucose transporter - 2 (SGLT-2) in clear cell renal carcinoma: possible prognostic implications. Int Braz J Urol. (2019) 45:169–78. doi: 10.1590/S1677-5538.IBJU.2018.0271 PMC6442141 30521176

[51] Torre LA Bray F Siegel RL Ferlay J Lortet-Tieulent J Jemal A. Global cancer statistics, 2012. CA Cancer J Clin. (2015) 65:87–108. doi: 10.3322/caac.21262 25651787

[52] Saito T Okada S Yamada E Shimoda Y Osaki A Tagaya Y. Effect of dapagliflozin on colon cancer cell [Rapid Communication. Endocr J. (2015) 62:1133–7. doi: 10.1507/endocrj.EJ15-0396 26522271

[53] Kato J Shirakami Y Ohnishi M Mizutani T Kubota M Sakai H. Suppressive effects of the sodium glucose cotransporter 2 inhibitor tofogliflozin on colorectal tumorigenesis in diabetic and obese mice. Oncol Rep. (2019) 42:2797–805. doi: 10.3892/or.2019.7357 31638239

[54] Chan RNC Chan RNF Chou OHI Tse G Lee S. Lower risks of incident colorectal cancer in SGLT2i users compared to DPP4i users: A propensity score-matched study with competing risk analysis. Eur J Intern Med. (2023) 110:125–7. doi: 10.1016/j.ejim.2023.01.021 36732129

[55] Tang H Dai Q Shi W Zhai S Song Y Han J. SGLT2 inhibitors and risk of cancer in type 2 diabetes: a systematic review and meta-analysis of randomised controlled trials. Diabetologia. (2017) 60:1862–72. doi: 10.1007/s00125-017-4370-8 28725912

[56] Jiang D Ma P. Canagliflozin, characterized as a HDAC6 inhibitor, inhibits gastric cancer metastasis. Front Oncol. (2022) 12:1057455. doi: 10.3389/fonc.2022.1057455 36457493 PMC9705739

[57] Okada J Matsumoto S Kaira K Saito T Yamada E Yokoo H. Sodium glucose cotransporter 2 inhibition combined with cetuximab significantly reduced tumor size and carcinoembryonic antigen level in colon cancer metastatic to liver. Clin Colorectal Cancer. (2018) 17:e45–8. doi: 10.1016/j.clcc.2017.09.005 29054804

[58] Shi N Shi Y Xu J Si Y Yang T Zhang M. SGLT-2i and risk of Malignancy in type 2 diabetes: A meta-analysis of randomized controlled trials. Front Public Health. (2021) 9:668368. doi: 10.3389/fpubh.2021.668368 34164370 PMC8215266

[59] Wang Y Yang L Mao L Zhang L Zhu Y Xu Y. SGLT2 inhibition restrains thyroid cancer growth via G1/S phase transition arrest and apoptosis mediated by DNA damage response signaling pathways. Cancer Cell Int. (2022) 22:74. doi: 10.1186/s12935-022-02496-z 35148777 PMC8840070

[60] Xie Z Wang F Lin L Duan S Liu X Li X. An SGLT2 inhibitor modulates SHH expression by activating AMPK to inhibit the migration and induce the apoptosis of cervical carcinoma cells. Cancer Lett. (2020) 495:200–10. doi: 10.1016/j.canlet.2020.09.005 32931885

[61] Wu W Zhang Z Jing D Huang X Ren D Shao Z. SGLT2 inhibitor activates the STING/IRF3/IFN-β pathway and induces immune infiltration in osteosarcoma. Cell Death Dis. (2022) 13:523. doi: 10.1038/s41419-022-04980-w 35662245 PMC9166744

[62] Nakachi S Okamoto S Tamaki K Nomura I Tomihama M Nishi Y. Impact of anti-diabetic sodium-glucose cotransporter 2 inhibitors on tumor growth of intractable hematological Malignancy in humans. BioMed Pharmacother. (2022) 149:112864. doi: 10.1016/j.biopha.2022.112864 35367765

[63] Shoda K Tsuji S Nakamura S Egashira Y Enomoto Y Nakayama N. Canagliflozin inhibits glioblastoma growth and proliferation by activating AMPK. Cell Mol Neurobiol. (2023) 43:879–92. doi: 10.1007/s10571-022-01221-8 PMC11415156 35435536

[64] Min J Wu L Liu Y Song G Deng Q Jin W. Empagliflozin attenuates trastuzumab-induced cardiotoxicity through suppression of DNA damage and ferroptosis. Life Sci. (2023) 312:121207. doi: 10.1016/j.lfs.2022.121207 36403642

[65] Ren C Sun K Zhang Y Hu Y Hu B Zhao J. Sodium-glucose coTransporter-2 inhibitor empagliflozin ameliorates sunitinib-induced cardiac dysfunction via regulation of AMPK-mTOR signaling pathway-mediated autophagy. Front Pharmacol. (2021) 12:664181. doi: 10.3389/fphar.2021.664181 33995090 PMC8116890

[66] Barış VÖ Dinçsoy AB Gedikli E Zırh S Müftüoğlu S Erdem A. Empagliflozin significantly prevents the doxorubicin-induced acute cardiotoxicity via non-antioxidant pathways. Cardiovasc Toxicol. (2021) 21:747–58. doi: 10.1007/s12012-021-09665-y 34089496

[67] Mahmoud Refaie MM Bayoumi AM Mokhemer SA Shehata S Abd-El-Hameed NM. Role of hypoxia inducible factor/vascular endothelial growth factor/endothelial nitric oxide synthase signaling pathway in mediating the cardioprotective effect of dapagliflozin in cyclophosphamide-induced cardiotoxicity. Hum Exp Toxicol. (2023) 42:9603271231193392. doi: 10.1177/09603271231193392 37526264

[68] Chang WT Lin YW Ho CH Chen ZC Liu PY Shih JY. Dapagliflozin suppresses ER stress and protects doxorubicin-induced cardiotoxicity in breast cancer patients. Arch Toxicol. (2021) 95:659–71. doi: 10.1007/s00204-020-02951-8 33211168

[69] Faggiano A Gherbesi E Cardinale D Vicenzi M Carugo S. SGLT2-i prevent left ventricular dysfunction induced by anthracycline in mouse model: A systematic-review and meta-analysis. Vascul Pharmacol. (2023) 150:107171. doi: 10.1016/j.vph.2023.107171 37061151

[70] Gongora CA Drobni ZD Quinaglia Araujo Costa Silva T Zafar A Gong J Zlotoff DA. Sodium-glucose co-transporter-2 inhibitors and cardiac outcomes among patients treated with anthracyclines. JACC Heart Fail. (2022) 10:559–67. doi: 10.1016/j.jchf.2022.03.006 PMC9638993 35902159

[71] Shi H Zeng Q Wei Y Yang H Tang H Wang D. Canagliflozin is a potential cardioprotective drug but exerts no significant effects on pirarubicin induced cardiotoxicity in rats. Mol Med Rep. (2021) 24:703. doi: 10.3892/mmr.2021.12342 34368866

[72] Abdelrahman AM Al Suleimani Y Shalaby A Ashique M Manoj P Nemmar A. Effect of canagliflozin, a sodium glucose co-transporter 2 inhibitor, on cisplatin-induced nephrotoxicity in mice. Naunyn Schmiedebergs Arch Pharmacol. (2019) 392:45–53. doi: 10.1007/s00210-018-1564-7 30206656

[73] Park CH Lee B Han M Rhee WJ Kwak MS Yoo TH. Canagliflozin protects against cisplatin-induced acute kidney injury by AMPK-mediated autophagy in renal proximal tubular cells. Cell Death Discovery. (2022) 8:12. doi: 10.1038/s41420-021-00801-9 35013111 PMC8748642

[74] Kabel AM Estfanous RS Alrobaian MM. Targeting oxidative stress, proinflammatory cytokines, apoptosis and toll like receptor 4 by empagliflozin to ameliorate bleomycin-induced lung fibrosis. Respir Physiol Neurobiol. (2020) 273:103316. doi: 10.1016/j.resp.2019.103316 31600583

[75] De Jonghe S Proctor J Vinken P Feyen B Wynant I Marien D. Carcinogenicity in rats of the SGLT2 inhibitor canagliflozin. Chem Biol Interact. (2014) 224:1–12. doi: 10.1016/j.cbi.2014.09.018 25289773

[76] De Jonghe S Johnson MD Mamidi RNVS Vinken P Feyen B Lammens G. Renal tubular and adrenal medullary tumors in the 2-year rat study with canagliflozin confirmed to be secondary to carbohydrate (glucose) malabsorption in the 15-month mechanistic rat study. Chem Biol Interact. (2017) 277:85–90. doi: 10.1016/j.cbi.2017.09.008 28916336

[77] Mamidi RN Proctor J De Jonghe S Feyen B Moesen E Vinken P. Carbohydrate malabsorption mechanism for tumor formation in rats treated with the SGLT2 inhibitor canagliflozin. Chem Biol Interact. (2014) 221:109–18. doi: 10.1016/j.cbi.2014.08.001 25130857

[78] Bogdanffy MS Stachlewitz RF van Tongeren S Knight B Sharp DE Ku W. Nonclinical safety of the sodium-glucose cotransporter 2 inhibitor empagliflozin. Int J Toxicol. (2014) 33:436–49. doi: 10.1177/1091581814551648 25260362

[79] Knight B Yuan J Koegler S Pande P Hall J Hill JD. Pathogenesis of renal injury and gene expression changes in the male CD-1 mouse associated with exposure to empagliflozin. Toxicol Pathol. (2018) 46:671–82. doi: 10.1177/0192623318784514 29945496

[80] Phillips JA Taub ME Bogdanffy MS Yuan J Knight B Smith JD. Mode of action and human relevance assessment of male CD-1 mouse renal adenocarcinoma associated with lifetime exposure to empagliflozin. J Appl Toxicol. (2022) 42:1570–84. doi: 10.1002/jat.4329 35393688

[81] Miller RA Harrison DE Allison DB Bogue M Debarba L Diaz V. Canagliflozin extends life span in genetically heterogeneous male but not female mice. JCI Insight. (2020) 5:e140019. doi: 10.1172/jci.insight.140019 32990681 PMC7710304

[82] Korfhage J Skinner ME Basu J Greenson JK Miller RA Lombard DB. Canagliflozin increases intestinal adenoma burden in female apcmin/+ Mice. J Gerontol A Biol Sci Med Sci. (2022) 77:215–20. doi: 10.1093/gerona/glab254 PMC8824675 34448851

